# The use and misuse of ratio and proportion exposure measures in food environment research

**DOI:** 10.1186/s12966-020-01019-1

**Published:** 2020-09-21

**Authors:** Lukar E. Thornton, Karen E. Lamb, Simon R. White

**Affiliations:** 1 Institute for Physical Activity and Nutrition (IPAN), School of Exercise and Nutrition Sciences, Deakin University, Burwood, Australia; 2 Melbourne School of Population and Global Health, The University of Melbourne, Parkville, Australia; 3 Medical Research Council (MRC) Biostatistics Unit, University of Cambridge, Cambridge, UK

**Keywords:** Food environment, Built environment, Neighbourhood, Dietary behaviours, Obesity

## Abstract

**Background:**

The food stores within residential environments are increasingly investigated as a possible mechanism driving food behaviours and health outcomes. Whilst increased emphasis is being placed on the type of study designs used and how we measure the outcomes, surprisingly little attention gets diverted to the measures of the food environment beyond calls for standardised approaches for food store coding and geographic scales of exposure. Food environments are a challenging concept to measure and model and the use of ratio and proportion measures are becoming more common in food environment research. Whilst these are seemingly an advance on single store type indicators, such as simply counting the number of supermarkets or fast food restaurants present, they have several limitations that do not appear to have been fully considered.

**Main body:**

In this article we report on five issues related to the use of ratio and proportion food environment measures: 1) binary categorisation of food stores; 2) whether they truly reflect a more or less healthy food environment; 3) issues with these measures not reflecting the quantity of food stores; 4) difficulties when no stores are present; and 5) complications in statistical treatment and interpretation of ratio and proportion measures. Each of these issues are underappreciated in the literature to date and highlight that ratio and proportion measures need to be treated with caution.

**Conclusion:**

Calls for the broader adoption of relative food environment measures may be misguided. Whilst we should continue to search for better ways to represent the complexity of food environments, ratio and proportion measures are unlikely to be the answer.

## Background

Over the last 20 years, researchers investigating the role of neighbourhood food environments on food behaviours and health outcomes have used an increasing number of different and, in appearance, more sophisticated measures to capture exposure to the food environment [1–7]. As an example, food environment exposure measures have shifted from linking exposure data at an administrative unit level (e.g., fast food restaurants within a postcode [8]), to GIS measures created around individual households (e.g., fast food restaurants within a buffer [9–11]), and more recently to capturing exposure within activity spaces (e.g., fast food restaurants within the daily paths travelled by an individual [12]). As debated elsewhere, there is no clear consensus on the most appropriate exposure measures and consequently this limits our ability to understand how the food environment influences health and behaviour [13–16].

The availability of more detailed food retail data has led to the development of various measures that account for the mix of food stores [17, 18]. Increasingly, such measure are utilising ratio and proportion indicators and the availability of such indicators, including the Modified Retail Food Environment Index [19], has likely contributed to their increased use. A ratio measure may consider the number of unhealthy food stores relative to healthy food stores [20–27] or vice versa [19, 28]. For example, if an individual is exposed to three unhealthy food stores and one healthy food store, the ratio of unhealthy food stores to healthy food stores for that individual is 3 (i.e., unhealthy/ healthy = ratio of 3/1). Commonly used proportion indicators measure the proportion of all food stores classified as either healthy [29–34] or unhealthy [7, 34, 35] or the proportion of all restaurants that are fast food restaurants [25, 26, 32, 36–39]. Again, if an individual is exposed to three unhealthy food stores and one healthy food store, the proportion of healthy stores is 0.25 (i.e., a healthy/total number of stores = 1/(1 + 3) = 1/4). Others have utilised a similar approach based on the densities of stores estimated via Kernel density estimates [40, 41] or other measures of spatial access [42]. In terms of their statistical treatment, in analyses ratio and proportion exposure measures are considered as these single values to represent the mix of stores within a food environment.

Whilst it is important to consider the totality of food retailers to get a sense of all options available to consumers, a simple ratio or proportion indicator may not adequately capture the complexity and intricacy of the different food stores available. In this commentary, we outline why these measures may be too simplistic and potentially misleading indicators of the food environment, highlighting the need for more methodological research into how to appropriately capture multiple aspects of the food environment.

### Categorisation of food stores

To calculate a ratio or proportion measure of the food environment it is first necessary to categorise the food store types.

#### Ratio

Ratios rely on binary categorisations and assigning all food stores into one of those two categories. As an example, in ratio measures stores are categorised as either healthy and unhealthy (or ‘less healthy’) [20–23, 25, 28]. Food store categorisation continues to be problematic [43, 44] and is not advanced by a push towards a binary categorisation of food stores. Food stores present in an area are likely to be excluded from a ratio measure if they do not neatly fit into the two classifications used. This means that the overall food environment is unlikely to be adequately captured using the ratio measures.

Whilst this issue is true of most summary measures of the food environment, we assert that crude binary categories used in ratio measures are particularly prone to substantial loss of differentiation. This reduces the ability to determine the potential role of the food environment in influencing food behaviours. A binary split of food stores considered as unhealthy (limited definition, e.g. excludes small candy stores, ice cream vendors)/healthy or unhealthy (more expansive definition)/healthy will give differing (but equally valid) ratios. As an example, consider a researcher using food store definitions such that an area has three unhealthy stores, two healthy stores, and three unclassified stores, dropping the unclassified stores giving a ratio of 3/2 = 1.5; but the more expansive definition might yield six unhealthy stores, two healthy stores, and no unclassified stores, giving a ratio of 6/2 = 3.

If we are satisfied with the categorisation of stores, then we can compare two areas; one area may have three convenience stores (categorised as unhealthy) and a large supermarket (categorised as healthy) (ratio [unhealthy/healthy] = 3) and whilst another has three large chain fast food restaurants (categorised as unhealthy) and one greengrocer (categorised as healthy) (ratio [unhealthy/healthy] = 3). Whilst the ratio indicator is the same in these two examples, the actual difference in the food environment is lost through the binary classification.

#### Proportion

Food store classifications are also problematic for proportion indicators. In many instances, the denominator of the proportion measure is the total number of food stores (n.b. commonly this is the sum of ‘healthy’ and ‘unhealthy’ food stores in a binary classification) [19, 25, 29–33, 35, 40, 41, 45, 46]. If the types of foods stores included in these categorisations are restrictive (e.g., healthy stores only include supermarkets and greengrocers, while unhealthy stores only include fast food restaurant and convenience stores), then numerous other stores selling food (e.g., fish mongers, bakeries, and others outlined by Lucan et al. [44]) will be excluded from the denominator. This has substantial implications for the proportion indicator (e.g., a limited measure [4 healthy food stores: 10 total food stores, proportion = 0.4] vs. a more comprehensive measure [4 healthy food stores: 20 total food stores, proportion = 0.2]). Whilst this may limit the comparability of studies, it is also important to note that the varied classifications may be appropriate depending on the study context and research question. It is therefore important that included (and excluded) store types are reported in detail. Tools exist to guide the reporting of food environment measures and should be more widely adopted [47].

### Ratio and proportion indicator may not necessarily reflect a healthy or unhealthy food environment

It is important to recognise that a lower ratio of healthy to unhealthy stores, or low proportion of healthy stores to all stores, may not necessarily reflect a food environment with inadequate opportunities to engage in healthy food behaviours. The example provided in Table 1 shows three hypothetical neighbourhoods. Assuming each neighbourhood has similar population numbers and socio-demographic characteristics, Area 1 is exposed to the healthiest food environment according to both the ratio and proportion measure even though the absence of a supermarket means residents have limited opportunities to source all weekly food requirements. In Area 2, residents may also find it difficult to source weekly food requirements, or perhaps high-quality and affordable fresh produce, with only one mid-size supermarket available in this neighbourhood. However, using a ratio or proportion measure, both Area 1 and Area 2 are considered healthier from a food environment perspective than Area 3. In Area 3, the large supermarket, fruit and vegetable market, and an ethnic grocer provide adequate opportunities for residents to source fresh produce and their weekly food requirements. To supplement access to these stores, the presence of two convenience stores provide opportunities to buy top up items such as bread and milk although the presence of energy-sense snack foods within such stores means they are often categorised as unhealthy (or ‘less healthy’) [19–23, 25, 28–31, 33, 35, 40, 41]. The examples provided here shows that a higher ratio of unhealthy to healthy stores does not necessarily mean that the stores present do not offer healthier food as was reported in an early definition of ratio measures [23]. Condensing these nuances of food environments to a single value reduces the ability to understand the true mix of food retail stores in the areas being considered. Whilst this is not isolated to the use of ratio and proportion indicators, the example provided demonstrates how these measures may be misleading.

**Table 1:** Food store classifications and the ratio and proportion values in three example neighbourhoods

	Unhealthy food stores	Healthy food stores	Ratio (unhealthy: healthy)	Proportion (healthy: all food stores)
**Area 1**	1 x chain fast food restaurant	2 x greengrocer (fruit and vegetable store)	0.5	0.66
**Area 2**	1 x small takeaway food store (e.g. independent pizzeria)	1 x mid-size supermarket (grocery store)	1	0.5
**Area 3**	1 x chain fast food restaurant 2 x convenience stores 2 x small takeaway food stores	1 x large supermarket 1 x fruit and vegetable market 1 x ethnic grocery store	1.67	0.375

### The quantity of food stores is not reflected in relative measures

#### Ratio

Ratio measures only consider relative quantities of stores, not the absolute quantities. Whilst this is known and often acknowledged by researchers using these measures and is indeed the impetus for choosing such measures, one of the key downsides is that they do not differentiate between areas that have low numbers of both unhealthy and healthy food stores and areas with high numbers of both unhealthy and healthy food stores. As demonstrated in Table 2, ratios can remain the same when both the number of unhealthy and healthy stores in an area increase. Further, in the example provided, there is clearly a greater disparity in the absolute number of unhealthy to healthy stores in Area 3 compared to Area 1 but again this is lost using a ratio measure. These issues are problematic as we should not expect the influence of the food environment to be the same on health and behaviour outcomes irrespective of the quantities of food stores. This is because a greater number of stores may increase accessibility through a higher likelihood of exposure along a residents chosen travel route, potentially longer opening hours amongst some stores which may benefits those working irregular hours, greater product variety across stores, and potentially more competitive prices.

**Table 2:** Similarities in ratio and proportion values when absolute number of stores vary

	Number of unhealthy food stores	Number of healthy food stores	Ratio (unhealthy: healthy)	Proportion (healthy: all food stores)
**Area 1**	2	1	2	0.33
**Area 2**	4	2	2	0.33
**Area 3**	8	4	2	0.33
	**Number of fast food restaurants**	**Number of full-service restaurants**	**Ratio (fast food: full-service restaurants)**	**Proportion (fast food: all restaurants)**
**Area 4**	1	1	1	0.5
**Area 5**	6	6	1	0.5

#### Proportion

The fundamental problem outlined above remains relevant for proportion measures. One common proportion measure used in the food environment literature is the number of fast food restaurants relative to all restaurants [32, 36–38]. It would not be unusual to find a less commercialised area with one fast food restaurant and one sit-down restaurant (two in total) and another more commercialised area with six fast food restaurant and six sit-down restaurants (twelve in total). However, in this instance the ratio value for both areas remains at 1 and the proportion value for both areas remains at 0.5. Prior research has found that variety of fast food restaurants is a potentially overlooked indicator [10] and thus the ability to explore this is lost when ratio and proportion measures are used.

### Problems when the category of either healthy of unhealthy stores contains a zero

In a situation where the numerator is zero (e.g. no unhealthy stores), both the ratio and proportion will be represented as a zero, regardless of the denominator value (e.g. number of healthy/total stores) assuming this is greater than zero (Table 3). It is unlikely that the effect of the food environment on health or behaviour is the same for each of these quite different food environments but if they are each treated as zero, ultimately that is what is being assumed. A further challenge with ratio measures is that a zero denominator results in an undefined estimate. This means that ratios of unhealthy to healthy food stores, for example, are undefined for areas with unhealthy food stores present but no healthy food stores (e.g., three unhealthy food stores/zero healthy food stores = undefined ratio). Finally, for both ratios and proportions, it is clearly problematic if there are no stores of any type present as again the indicator would be an undefined estimate.

**Table 3:** Ratio and proportion values when the numerator or denominator value contains a zero

	Number of unhealthy food stores (numerator)	Number of healthy food stores (ratio denominator)	Ratio (unhealthy: healthy)	Proportion (unhealthy: all food stores)
**Area 1**	0	1	0	0
**Area 2**	0	3	0	0
**Area 3**	1	0	Undefined	1
**Area 4**	7	0	Undefined	1
**Area 5**	0	0	Undefined	Undefined

#### Observed methods for dealing with zero values

Researchers using ratio or proportion measures are faced with some difficult decisions regarding how to handle zero values in either the numerator, the denominator, or both. From the articles assessed, it was not always possible to tell if zeroes were present and, if so, how these were treated in the statistical analysis. One option was to omit individuals from all or at least part of the analysis if they had a zero value for the denominator [25, 33, 36–38, 46] although some of these studies ran analysis with and without zeroes where appropriate. An alternative approach is to add some value, such as one [21], to the denominator if it was a zero. This enables the ratio to be calculated by treating areas with no stores in the denominator category as if they had one. Again, this approach is less than satisfactory given that areas with two unhealthy food stores and one healthy food store will be assigned the same ratio as those with two unhealthy food stores and no healthy food stores. A third option is to create a separate category in which there is a zero denominator to indicate that there are no stores present [29, 48]. This approach allows all data to be used but then results in a situation in which the continuous ratio or proportion exposure has to be used as a categorical exposure and thus some arbitrary choice of cut-point may need to be made to categorise these. This can result in a loss of power to detect associations, among other concerns [49].

### Statistical treatment and interpretation

A further challenge in dealing with ratio or proportion measures is deciding how to treat these in statistical analyses. Both are continuous positive food environment exposure measures, and although the proportion is limited in range from zero to one, it could plausibly be used as continuous variables in a statistical model and studies have indeed treated it this way [25, 28, 32, 38, 50]. However, one challenge in using ratio measures is how to interpret the coefficients in the regression models. For example, suppose a coefficient of 0.1 is obtained when examining a model of the unhealthy to healthy food ratio as an exposure for weight in kilograms as the outcome. This means that, on average, weight increases by 0.1 kg with each unit increase in the ratio of unhealthy to healthy stores, holding all other variables in the model constant. This suggests that it may be good to increase the number of healthy stores relative to the number of unhealthy stores but provides no information about the quantities of stores that have an influence on weight. Ultimately, the ratio measures provide limited information on what makes a food environment adequate to ensure positive health effects.

Another concern related to using ratio or proportion measures is that there may be non-linear relationships between these food environment exposures and the health or behaviour outcomes which are not appropriately taken into account when simply including the measure as a continuous exposure or categorising the exposure arbitrarily. While research has examined the shape of the relationship in the proportion of health-harming food stores of all food stores and health, specifically the odds of Type II diabetes [35], the interpretation of the findings remained challenging. Mezuk et al. (2016) found a curvilinear relationship and noted that this implies areas with limited access to any stores and areas with high proportions of health-harming stores are both associated with higher levels of Type II diabetes [35]. These findings suggest that, should ratio or proportion measures be adopted, relationships may have to be modelled in a more complex manner. However, as mentioned, this adds to greater complexity in interpreting the model coefficients in a meaningful way to understand how the food environment influences health or behaviour. Categorisation of the ratio or proportion exposure has been used elsewhere [21, 29, 30, 48]. This approach may appear appealing as it can assist in dealing with non-linear relationships and seems to make interpretation of the model coefficients easier (e.g., studies could describe the difference in mean outcome between ‘low’ or ‘high’ ratios). However, the reasons behind the choice of categories are often unclear, with some studies adopting percentile categorisation, such as tertiles [31]. These data driven approaches to categorisation can be problematic, resulting in challenges in comparing findings across studies [49] particularly when values for the category range are not specified so it is unclear what low and high ratios or proportions represent in the study.

## Conclusion

Although a recent Australian study noted “ratio-based measures of healthy to unhealthy food stores have been rarely investigated” ( [48] p.103), it appears that the use of ratio and proportion measures are in fact prominent in the food environment field with a full systematic search of the literature likely to reveal even more studies than those already cited in this debate. Whilst it is fully acknowledged that measures are needed that capture the mix of food retail stores available, recent calls to advocate for ratio and proportion measures may be misguided [7, 26, 41, 51, 52].

Beyond the ratio and proportion measures discussed here, a measure that weights different store types by their potential contribution to healthy and unhealthy food behaviours whilst also factoring in the quantity of stores has been developed to represent the totality of the food environment [17]. While appealing as this offers a more comprehensive overview of the food environment than those captured by ratios or proportions, this measure may suffer from issues related to multicollinearity [53] due to the healthy and unhealthy stores being treated as separate variables as some areas may have either high numbers or low numbers of both healthy and unhealthy stores. Whilst this measure differentiates between stores types (e.g. large supermarket vs small supermarket), it suffers from treating all stores within a category (e.g. a convenience store) the same irrespective of specific variations in the products available. Additionally, it is noted that this food environment score and many other measures are typically limited to residential environments and calls for assessments of more personalised exposures have previously been published [54–56].

It is recognised that some (not all) of the issues raised hold true for absolute quantity measures. However, one of the unique points about ratio and proportion measures is that they are advocated for as an advance on absolute quantity measures and often viewed as being more sophisticated [26, 51, 52]. As demonstrated, this is potentially not the case and thus this debate paper makes an important contribution to the literature by highlighting these issues with the aim of redirecting the collective focus of this field. Whilst the ultimate solution may not yet exist, food environment researchers should work collectively towards developing more sophisticated approaches to food retail mix that move beyond ratios and proportions rather than accept the limitations of existing measures.

## Data Availability

Not applicable.
